# E-mail-based health care in patients with dementia during the pandemic

**DOI:** 10.3389/fpsyt.2022.863923

**Published:** 2022-08-08

**Authors:** Kubra Altunkalem Seydi, Esra Ates Bulut, Idil Yavuz, Hemrin Kavak, Derya Kaya, Ahmet Turan Isik

**Affiliations:** ^1^Unit for Aging Brain and Dementia, Department of Geriatric Medicine, Faculty of Medicine, Dokuz Eylul University, Izmir, Turkey; ^2^Geriatric Science Association, Izmir, Turkey; ^3^Department of Geriatric Medicine, Adana City Training and Research Hospital, Adana, Turkey; ^4^Department of Statistics, Faculty of Science, Dokuz Eylul University, Izmir, Turkey

**Keywords:** older adults, dementia, telemedicine, information technology, remote consultation

## Abstract

**Introduction/aim:**

Frail and cognitively impaired older patients are particularly vulnerable groups during the pandemic. Lockdowns, social isolation, and physical inactivity considerably affect physical and mental wellbeing. During the pandemic process, routine medical checks and acute medical care services may be disrupted. The study aimed to demonstrate the feasibility and effectiveness of telemedicine in the delivery of healthcare services to elderly patients during the pandemic.

**Materials and methods:**

E-mails sent to the e-mail address of the department of geriatrics, which has been actively used for 4 years, between April 2020 and June 2021, were retrospectively evaluated. The time and reason for each application, referral to the patients, demographic data of the patients, and chronic diseases were recorded. E-mail frequencies were considered monthly time series, and time series charts for e-mail frequencies from patients were produced.

**Results:**

A total of 374 e-mails that 213 patients sent were assessed. A vast majority, 97.6% of the e-mails, were sent by proxies. The mean age of patients was 78.7 ± 8.1 years, and 59.2% were women. Hypertension and dementia were the most common comorbidities. The applications mostly occurred in April-May and October-November 2020. The most common complaint in dementia was behavioral disturbances (13.6%). Geriatric outpatient appointments were arranged for 29.9% of the applicants, 14.2% were referred to the emergency department, and 23.0% were offered medical treatment. Outpatient examination and treatment were completed in 15% of the patients and 10.4% of them were hospitalized. The time series charts showed that e-mails were sent more frequently by patients with dementia than the others (*p* = 0.03).

**Conclusions:**

Telemedicine, which enables many problems of patients to be solved in geriatric practice without face-to-face appointments, can also prevent infections and unnecessary hospitalizations, especially during these unusual pandemic days.

## Introduction

COVID-19, which emerged in China in 2019, quickly spread all over the world and became a pandemic in a short time, creating a serious problem in terms of transmission to health personnel in health institutions and especially to patients in the risk group who go to the health institutions to receive health services (1). Elderly patients who are frail and whose cognitive functions are affected represent the group most associated with poor health outcomes of the pandemic. Quarantines, social isolation, and physical inactivity significantly affect physical and mental health (2). In particular, the restrictions implemented to maintain the social distance necessary to prevent the spread of the epidemic are some of the important changes that the epidemic brought to our lives. Elderly patients with multiple comorbidities may not show the typical symptoms of pulmonary infections such as fever, cough, and chest pain as younger people do. They may present with atypical presentations such as confusion, acute mental changes, frequent falls, movement disorder, loss of appetite, dysphagia, and incontinence (3). Due to the chronic changes caused by biological aging in elderly individuals, comorbidities are more common, and nutritional deficiencies, decreased effectiveness of the mucosal barrier, and atypical symptoms of infection make it difficult to understand and treat infections (4, 5). Due to the changes that occur in both the immune system and other physiological organ systems against pathogens with advancing age, infectious diseases are more common in geriatric patients and these diseases may progress more severely than in young people (5). The fact that the elderly population is at high risk for COVID-19 has brought along the search for regulations to protect elderly individuals. As in the whole world, there have been various restrictions applied more strictly to the elderly due to the COVID-19 pandemic in our country, and various disruptions have been experienced in the applications of patients to health institutions due to routine controls. Therefore, telemedicine, which reduces the risk of transmission by isolating patients during the pandemic period, and also provides service *via* technological communication tools (such as e-mail, and video conference) to maintain the continuity of health care, has come to the fore (6). Telemedicine is the rapid access to remote medical experience and information using telecommunication and information technologies (7). It involves the use of various types of information and communication technologies (ICTs), such as computers, the Internet, and cell phones according to the World Health Organization (WHO) definition (8). Telemedicine can also be applied as an appropriate, safe, effective, and new method in clinical care in health-related emergencies (9).

The extraordinary burden on the healthcare system due to the pandemic leads healthcare providers to use telemedicine so that patients without a diagnosis of COVID-19 may benefit from healthcare services. This practice, which has shown a striking development in recent years and helps to perform periodic health checks in patient groups with chronic diseases by using limited personnel and financial resources, has become one of the more commonly used methods for the maintenance of health services without disruption during the pandemic period (10). During the period when many polyclinics are closed to face-to-face visits due to the restriction rules, it is possible to reach the patients at home, ensure the safety of other patients by maintaining social distance, and maintain the quarantine through telemedicine. Teleisolation application improves palliative care and patient support services, contributes to personnel safety and reduces the psychological burden caused by isolation, increases patient comfort in patients who are placed in isolation in the emergency department (11). Telemedicine service is implemented in various ways, among which, e-mail application is one of the methods that provide communication between healthcare personnel and patients (12). This study aimed to investigate the contribution of e-mail, which is used as a telemedicine service in elderly patients, to the realization of health care effectively and efficiently.

## Materials and methods

In our study, 374 e-mails were sent to the e-mail address of the Department of Geriatrics (geriatridanisma@deu.edu.tr) from a total of 213 patients between April 2020 and June 2021 and were evaluated retrospectively. The e-mail-based telemedicine service has been actively used for 4 years. Patients who were admitted to the outpatient clinics were informed about the e-mail service and suggested to contact the medical team in case of need. The e-mail account was checked every day by the geriatric fellow on-call. Each e-mail was replied to by the doctor's team within 48 h. Repetitive e-mails about the same patient within a week were not included in the study. The patients who utilized the e-mail technology by proxy were identified. The person who sent the e-mail, the complaint sent by e-mail, the referral given to the patient upon the e-mail response, the health condition that caused the patient to send the e-mail, the outpatient treatment or service hospitalization status of the patients who were summoned to the polyclinic control were recorded. The referrals given to the patients were determined as non-pharmacological intervention, drug regulation, geriatric outpatient control, emergency or another branch consultation deemed necessary. Other than these, appointment revision, drug refills, and control laboratory results evaluation were gathered under the “other” heading. After the evaluation of the patients who were hospitalized after their admission to the outpatient clinic, conditions that caused the need for inpatient treatment were grouped into disease progression, acute organ damage, COVID-19 or other infections, and adverse drug reactions. From the files of the patients included in the study, their demographic data, chronic diseases, diagnosis of dementia, and the drugs they used were examined.

### Statistical analysis

Statistical evaluation of the data was carried out using the IBM SPSS 25 program. Descriptive statistics were presented as mean ± standard deviation for continuous variables and as % for categorical variables. E-mail frequencies were considered as monthly time series and time series charts were generated for e-mail frequencies from patients. The relationship between patients' recurrent applications to telemedicine and their complaints were evaluated by regression analysis.

### Ethical approval

Ethical approval of the study was obtained on 08.09.2021 based on the decision numbered 2021/25-03 of the Non-Interventional Research Ethics Committee of our hospital. The study was conducted in accordance with the Declaration of Helsinki Principles.

## Results

The total number of patients who sent e-mails was 213. Of these patients, 88 had repeated admissions. The mean age of the 213 patients who applied was 78.74 ±8.13 years, 59.2% were women, 53.1% were married, and the rate of those who completed at least 8 years of education was calculated as 32.9%.

According to the records, approximately 1,200 patients were regularly actively followed up in our department during these 2 years (2019–2021), and the rate of e-mail usage was found to be 17%. In patients who benefited from telemedicine, hypertension and dementia were the most common comorbidities, respectively. When the accompanying comorbidities and sociodemographic characteristics of single applicants and recurrent applicants were examined, it was determined that there was no statistically significant difference (Table 1).

**Table 1:** Sociodemographic data and comorbidities of the patients by admission status.

	**Recurrent**	**Single**	***p***
	**Application**	**Application**	
	***n*: 88**	***n*: 125**	
Age	79.08 ± 7.78	78.44 ± 8.34	0.34
**Education (%)**			0.68
Illiterate	9.6	6.4	
Primary school	42.5	36.4	
High school	13.7	20.9	
University	19.2	20.9	
Sex (Female) (%)	58.0	60.0	0.76
**Marital status (%)**			0.97
Married	61.5	60.0	
Widow	15.4	15.5	
Divorced	23.1	24.5	
**Housing**			0.09
Alone	7.3	16.2	
With spouse	52.5	55.9	
With family	31.7	24.3	
With caregiver	8.5	2.7	
In an institution	0	0.9	
**Comorbidities (%)**			
Hypertension	62.5	57.3	0.44
Coronary artery disease	30.7	21.0	0.10
Cerebrovascular disease	3.4	8.9	0.11
Diabetes Mellitus	30.7	36.3	0.39
Dementia	56.8	45.2	0.09

In the evaluation of 374 e-mails received, it was observed that the most common reason for the application was behavioral disorder due to dementia. Due to the cancellation of routine outpatient clinic appointments in line with the restrictions proposed during the pandemic period, new appointment requests were in the second rank. These were followed by urinary system complaints, balance disorder, high blood pressure, pain, glycemic dysregulation, sleep and nutrition disorders, COVID-19 recommendations, acute mental change, cough, fever, and falls (Table 2). The recommendations given for the applications made by the patients were gathered under four main headings and others. The most common referral was geriatrics outpatient control recommendation (29.9%). Medication revisions were made by e-mail in 23% of all applications, 14.2% were referred to the emergency service or another related branch, and non-pharmacological recommendations were made to 19%. Outpatient examination and treatment were completed in 15% of the patients who were referred to the geriatric outpatient clinic, but 10.4% were hospitalized. It was observed that the most common disease progression (50%) was in the final diagnosis of the patients in the inpatient service. Infection, especially pneumonia, acute organ damage, and drug side effects were found to be other common causes of hospitalization (Table 2).

**Table 2:** Reasons for application.

**Applications**	**%**
***N*: 374**	
**By proxy**	97,6
**Causes**	
Fever	1.6
Pain	5.3
Cough	1.9
Acute mental status change	2.1
Imbalance	6.1
Sleep disturbance	4.8
Behavioral disturbances	13.6
Nutritional impairment	4.3
Urinary system complaints	6.4
Falls	1.3
Glycemic dysregulation	5.1
Blood pressure increase	5.3
Appointment cancellation	11.2
Others	27.8
COVID-19 information request	2.9
**Hospitalization**	10.4
**Referral**	
Non-pharmacological intervention	19.0
Drug regulation	23.0
Geriatric outpatient clinic appointment	29.9
Emergency referral	14.2
Others	13.9
**Outpatient application**	15.0
**Hospitalization**	10.4
Infection	22.2
COVID-19	2.8
Disease progression	50.0
Acute organ injury	13.9
Adverse drug reaction	11.1

**Figure 1:**
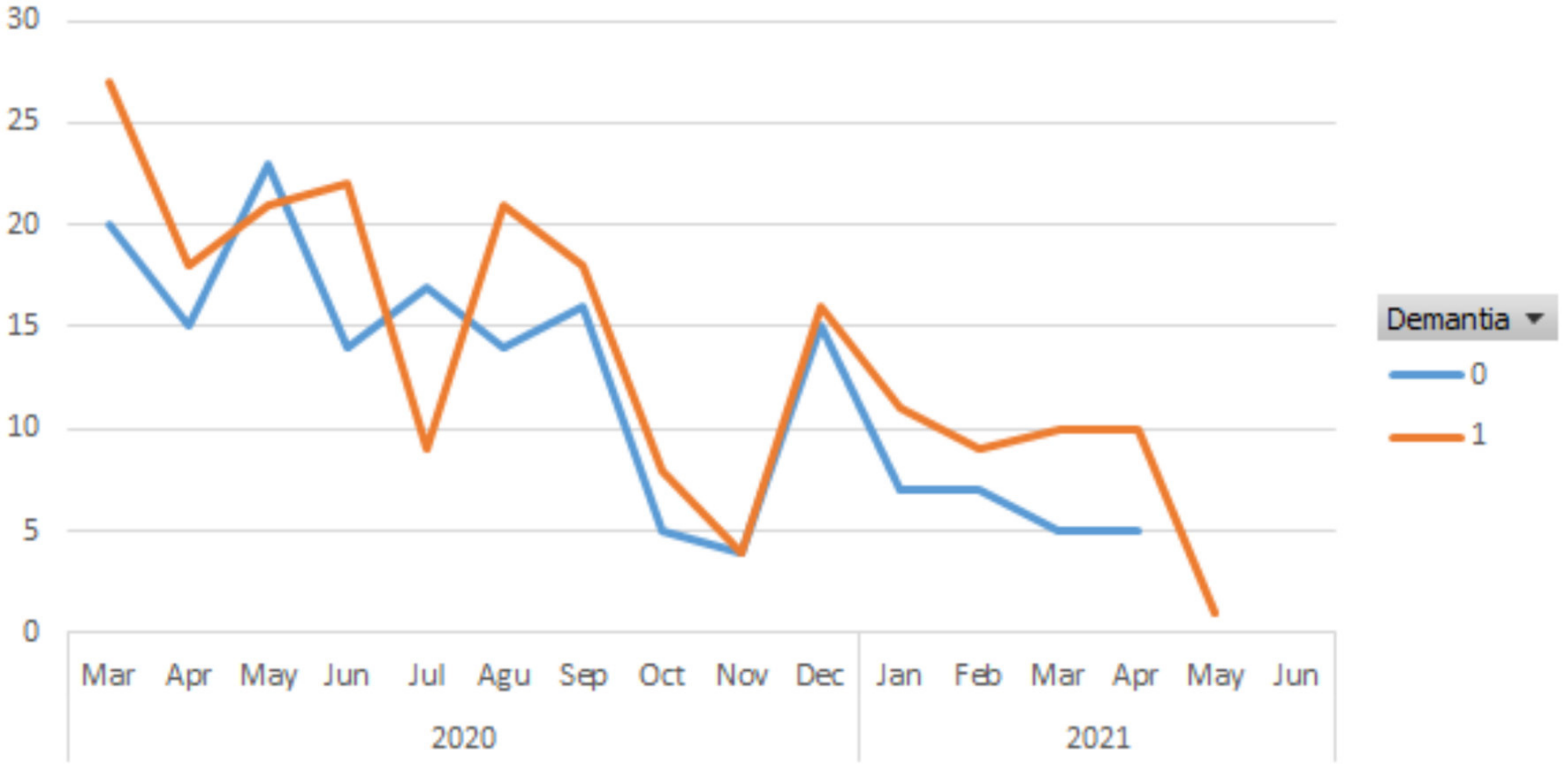
The time series plot of e-mails according to the months.

No statistically significant finding was found in the regression analysis performed to show whether there is a relationship between recurrent admissions and the reasons for admission (Table 3). When the monthly number of e-mail applications was evaluated, it was found that dementia patients applied more frequently and the applications were mostly in April-May and October-November, when the pandemic peaked in our country (Figure 1).

**Table 3:** The relationship between the reasons for application and the recurrent application.

**Variable**	**Beta**	**OR (CI)**	***p*-value**
Fever	0.138	1.148 (0.205–6.437)	0.875
Pain	−0.150	0.861 (0.335–2.214)	0.861
Cough	0.361	1.435 (0.271–7.603)	0.671
Gait and balance problems	−0.113	0.893 (0.366–2.175)	0.803
Sleep disturbance	−0.332	0.717 (0.270–1.907)	0.506
Behavioral disturbances	0.517	1.678 (0.834–3.374)	0.147
Urinary system complaints	1.054	2.870 (0.941–8.754)	0.064
Falls	−1.942	0.143 (0.016–1.311)	0.085
Glycemic dysregulation	0.474	1.607 (0.554–4.662)	0.383
Blood pressure increase	0.831	2.296 (0.737–7.154)	0.152

## Discussion

In this study investigating the frequency and effectiveness of telemedicine use *via* e-mail in the geriatric clinic, it was seen that the rate of telemedicine use among the patients followed up was approximately 17%, and patients commonly used this method because of dementia-related behavioral disorders. Because of the suggestions made to 55.9% of the incoming e-mails, it was observed that the existing medical problems of the patients were resolved before they came to the hospital during the peak times of COVID-19 and that the patients in need of acute care were directed to the inpatient service or the emergency room. Considering the recurrent applications, the most common reason for admission was behavioral disorder, appointment cancellation, and urinary system infection, as in single applications. It was remarkable that the majority of the applicants by e-mail belonged to dementia patients.

Thanks to telemedicine applications, which have become popular especially with the pandemic, for the increasing elderly population, the quality of life of individuals with chronic disease is improved by monitoring of the elderly at home, and the rate of admission to the hospital of the elderly is reduced by the use of information and communication technologies (13). The popularity of telemedicine applications increased gradually during the COVID-19 pandemic period, and many studies on the use of telemedicine in this process have been added to the literature (14). Especially in this period, telemedicine provided the possibility of maintaining social distance and reducing the risk of exposure to infection, namely for the high-risk population, while providing the opportunity for chronic disease management (15). Chronic diseases represent a major public health problem worldwide and are the leading cause of death among older adults (16). It has been shown that e-health services, which are used as communication technology, are both helpful and cost-effective in the diagnosis and management of chronic diseases such as congestive heart failure, stroke, chronic obstructive pulmonary disorder (COPD), diabetes mellitus (DM), hypertension, asthma, dementia and depression (17). As in our clinic, elderly patients with follow-up should be evaluated closely due to their accompanying comorbidities, multiple drug treatments, and geriatric syndromes (18). In the present study, it was observed that 59.2% of 213 patients who applied by e-mail had hypertension and 33.3% had DM. Of the total e-mail applications, 5.1% were for blood pressure and 5.3% for glycemic regulation. This supports the view that the dramatic improvement in telemedicine patient groups with chronic diseases helps to perform periodic health checks by using limited personnel and financial resources (10). In the period when many polyclinics are closed to face-to-face visits due to the restriction rules of patients, the continuity of health services is possible with telemedicine facilities by maintaining social distance, ensuring the safety of patients, and maintaining isolation (19). In our study, although geriatric outpatient control was recommended most frequently in the referrals given to the patients by e-mail, the continuation of the isolation was ensured by the home treatment arrangement of 23% of the patients. Non-pharmacological recommendations were made to 19% of the patients, 14.2% of whom were directed to related branches or emergencies, thus contributing to the prevention of unnecessary patient admissions and economic burden. There is research showing that quality virtual visits can improve the outcome of home care patients at a lower cost than traditional face-to-face home health visits, supporting the results in our clinic (20).

In the literature review in which telehealth and digital care types and applications are compiled for the current policies for COVID-19; health officials and policymakers are urged to consider social, organizational, and technological determinants to encourage the adoption of these practices for the current pandemic and future disasters (21). It was reported that the reasons for using telemedicine during the COVID-19 epidemic differ between studies, and common uses include clinical care, follow-up, medical education, diagnosis, rehabilitation, triage, research, surveillance, and contact tracing. Most of the studies in this field have been done on internal diseases and branches (14). Follow-up of the patients can be done *via* phone calls, video chat, and e-mail. In a retrospective cohort study conducted by Ramasmawy et al., the response status of healthcare providers in New York, which is considered to be the international epicenter of the COVID-19 pandemic, was evaluated with video consultations without face-to-face meetings, and it was concluded that telemedicine application as an alternative method to traditional clinical evaluation and patient satisfaction was high (22). Our study showed how effectively the e-mail system, which has been used for about 4 years in our geriatrics department to reduce the frequency of patients visiting the health center and to provide guidance in emergencies, is used especially during the pandemic period. When the monthly number of e-mail applications was evaluated, it was found that patients with dementia applied more frequently and the applications were mostly in April-May and October-November, when the pandemic peaked in our country. Organizing the appointments of the dementia patient and supporting basic and instrumental life activities are both a burden and a source of stress for the caregiver. It was determined that 49.8% of the 213 patients who applied and 56.8% of the 88 patients who applied recurrently had a diagnosis of dementia. Among the total e-mails, it was indicated that the most common reason for referral was behavioral disorder in dementia patients (13.6%). This result was similar when recurrent applicants were evaluated separately. However, no statistically significant finding was found in the regression analysis performed to show whether there is a relationship between recurrent admissions and the reasons for admission. The behavioral disorder occurring in dementia patients is characterized by rapid cognitive decline, low quality of life, and increased caregiver burden (23). Telemedicine caregiver assessment can reach caregivers where they are, providing needed support and guidance. Caregiver burden assessment scales may be applied face-to-face as well as in telemedicine visits by verbal and screen sharing methods (24). Rapidly adapting the telemedicine program, which has been carried out with 36 nursing homes since 2018 and introducing new protocols for COVID-19, Cormi et al. stated that hospitals in France, received 15 times more teleconsultation on March 1–28, 2020 compared to February, were prevented from becoming more crowded due to the applications (25). In the University of Rochester Specialized Oncology Care and Research in the Elderly clinic, telemedicine consultancy service has been implemented as the fastest and most efficient way of intervention to reduce the exposure of elderly and oncological patients to COVID-19 due to hospital admission. This application was evaluated as promising, but the inability to make eye contact during telephone visits, the absence of face-to-face interaction, and the inability to evaluate objective physical function were shown as disadvantages (26).

This has led healthcare providers to more advanced methods such as video conferencing. Thus, it has been stated that remote consultation by a trained health worker can be provided for a detailed geriatric evaluation (27).

Similarly, in our study, the inability to perform a physical examination, which is the cornerstone of detailed geriatric evaluation in our patients, and the accompanying physical problems such as vision-hearing impairment in many of our patients were the main difficulties we encountered in the practice of telemedicine. The fact that the elderly do not prefer to use the internet or cannot gain experience because they do not use technology, causes them to be deprived of digital services (28). In the study evaluating the relationship between telemedicine use and sociodemographic characteristics, participants classified as ready for telemedicine use were shown as married women, individuals younger than 80 years of age, Non-Hispanic Caucasians, those with at least a university degree, and those without myocardial infarction, DM, cancer, anxiety, and depressive states (28). Lam et al., in their study investigating the reasons why 4,525 adults with an average age of 79.6 were unprepared for telemedicine, stated that 38% were not ready for video visits due to their technological inexperience (29). Similarly, the fact that our patients are not a group that actively uses the internet and technology is a fundamental limiting factor in our country. However; In the period when the pandemic peaked in our country and quarantine measures were increased, the rate of the e-mail was found to be significantly higher than in other months. Considering that dementia patients constituted the most e-mailed group in this period, the benefit of the application is understood more clearly.

As far as we are concerned, our unit is one of the few centers where telemedicine is used effectively in elderly patients in our country. Follow-up of patients *via* e-mail has been carried out actively for about 4 years. In this respect, our study shows the characteristics of a pilot study. The limitation of the study is that it is retrospective. In addition, in this study, which was performed in a memory clinic with a reference center, the fact that patients may have a higher incidence of dementia may affect the generalizability of the study results.

## Conclusion

In terms of mortality and morbidity, due to COVID-19, older patients in the high-risk group can contact the clinic *via* their e-mail address for reasons such as their current clinical status and treatment recommendations, it is possible to guide patients, to ensure the safety of our patients by maintaining social distance, to comply with the isolation recommendations and to contribute to the protection of public health. Findings from this study reveal the importance of a practical telemedicine method with applicability and adoption during the pandemic in older adults. Telemedicine also enables regular follow-up to patients living in remote/rural areas not only during the pandemic period but also in ordinary circumstances. At the same time, this study's results encourage the healthcare personnel about the potential of digital technologies that provide clinical and psychological support during the pandemic and beyond. In addition to being a method that can be used effectively in elderly patients during the pandemic period, telemedicine also provides health services for patients who live far from health care or who have mobility/transport limitations. More studies are needed on the factors affecting the application of telemedicine in the provision of clinical services, its importance, and its replacement with clinical evaluation.

## Data availability statement

The datasets used and/or analyzed during the current study are available from the corresponding author on reasonable request.

## Ethics statement

Ethical approval of the study was obtained on 08.09.2021 based on the decision numbered 2021/25-03 of the Non-Interventional Research Ethics Committee of Dokuz Eylul University. The patients/participants provided their written informed consent to participate in this study.

## Author contributions

ATI and EAB made the study concept and design. KAS and HK helped in acquisition of data. IY performed analysis and interpretation of data. KAS and DK drafted the manuscript. ATI established critical revision of the manuscript for important intellectual content. All authors contributed to the article and approved the submitted version.

## Conflict of interest

The authors declare that the research was conducted in the absence of any commercial or financial relationships that could be construed as a potential conflict of interest.

## Publisher's note

All claims expressed in this article are solely those of the authors and do not necessarily represent those of their affiliated organizations, or those of the publisher, the editors and the reviewers. Any product that may be evaluated in this article, or claim that may be made by its manufacturer, is not guaranteed or endorsed by the publisher.
